# Longitudinal Shear in Timber–Concrete Composites with Flexible Adhesive Connections—Experimental and Numerical Investigations

**DOI:** 10.3390/ma17246055

**Published:** 2024-12-11

**Authors:** Klaudia Śliwa-Wieczorek, Armando La Scala, Wit Derkowski, Eva Binder

**Affiliations:** 1 Division of Bridge, Metal and Timber Structures, Faculty of Civil Engineering, Cracow University of Technology, 31-155 Cracow, Poland; 2 Dipartimento di Architettura Costruzione e Design, Polytechnic University of Bari, 70126 Bari, Italy; armando.lascala@poliba.it; 3 Division of Reinforced Concrete and Prestressed Concrete Structures, Faculty of Civil Engineering, Cracow University of Technology, 31-155 Cracow, Poland; wit.derkowski@pk.edu.pl; 4 The Building Technology Department, Linnæus University, 352 52 Växjö, Sweden; eva.binder@lnu.se

**Keywords:** shear, timber–concrete-composites, flexible adhesive, load–slip curve, stiffness, numerical analysis

## Abstract

Timber–concrete composites are established structural elements to combine the advantageous properties of both materials by connecting them. In this work, an innovative flexible adhesive connection in different configurations is investigated. Load-bearing capacity, stiffness, and the failure modes were first experimentally investigated by performing push-out tests. Subsequently, a numerical evaluation using ABAQUS 2017/Standard software was carried out in order to develop a three-dimensional numerical model. The Cohesive Zone Model (CZM) is employed to represent the adhesive characteristics at the contact areas between the Cross-Laminated Timber (CLT) and concrete elements. Three different connection configurations were evaluated, each consisting of five push-out specimens. The study investigates the impact of bonding surface area and the alignment of prefabricated glue strips with the load direction on the connection’s longitudinal shear load-bearing capacity, stiffness, and slip modulus. In addition, the impact of cyclic loads and the impact of time on displacements were analyzed. The average load capacity of the full surface connection (type A) is 44.5% and 46.2% higher than the vertical adhesive strips (type B) and the horizontal adhesive strips (type C), respectively. However, the initial stiffness of the tested joints depends on the orientation of the prefabricated adhesive fasteners, being approximately 20% higher when the bonding elements are aligned parallel to the load direction compared to when they are oriented perpendicularly.

## 1. Introduction

In recent years, there has been a strong emphasis on introducing changes in the construction industry to reduce its negative impact on the environment. A building’s carbon footprint and embodied energy can be reduced by replacing carbon-intensive materials, like concrete and steel, with wood [1]. Timber–Concrete Composites (TCC) are an example of a successful combination of two materials to achieve superior performance than the individual materials can provide. To achieve this, it is crucial to use connections that ensure composite action in cross-sections. There are several connecting methods available in timber engineering: traditional force transmission by direct contact between elements by grooved [2] or notched connections [3]; mechanical steel fasteners such as screws [4], steel dowels [5] or steel plates [6]; load transmission by adhesive bonding [7]; and combining glued and mechanical connections called hybrid connections [8,9].

Adhesive joints offer numerous advantages over traditional joining techniques, including more uniform stress distribution, energy dissipation, reduced stress concentration [10,11], enhanced fatigue resistance, sealing capabilities, acoustic insulation [12], vibration damping [13], and structural weight reduction. Furthermore, they exhibit higher stiffness, which is of particular importance when the governing factor is the serviceability limit state [10,14]. However, in the case of rigid adhesives, they also exhibit drawbacks such as limited resistance to high temperatures [15,16], sensitivity to increase in moisture content [17], low resistance to peel stresses [18,19], or durability problems [20].

Short-term tests at the University of Kassel by Schäfers and Seim [21] showed that bonding technology using epoxy adhesives (Sikadur 330) is convenient for bonding timber and Ultra-High Performance Concrete (UPHC). Researchers examined the influence of the material (three types of wood: spruce, beech, and unidirectional laminated veneer lumber), the influence of the surface treatment of concrete (sandblasted and ground), and the influence of different bond lengths on the behavior of the joint. Preliminary investigations indicated that the thickness of the bond-line and the surface treatment of timber have no influence on the bond strength [22]. They observed that the failure of the bond occurs in timber close to the bond-line in most cases. The surface treatment of UHPC does not affect the bond behavior. Regarding the type of timber, the beech specimens exhibited significantly higher ultimate loads compared to the other specimens. Moreover, they observed that the ultimate loads do not increase from a bond length of about 400 mm. A continuation of the research was work [23] on the long-term behavior for glued joints. The hygrothermal influence on the evolution of the bond strength and the time-dependent deformation was investigated with shear and bond specimens. Neither a decrease in the stiffness over time nor an increasing ratio of cohesive failure of the adhesive was detected. It was also found that the viscoelastic and mechano-sorbent material properties of wood have the greatest influence on the long-term behavior of the elements. The long-term studies by Tannert et al. [24] revealed that the adhesive bonding connection is a great way to achieve composite action between timber and concrete, while short-term investigations pointed to a linear behavior of the connection until load levels approach failure, with the failure occurring in the concrete in a brittle way. Giv et al. [25] investigated the effect of adhesive amount and type, i.e., polyurethane and epoxy, on the bond strength of wet and dry timber–concrete joints. It was found that epoxy-bonded wet TCC joints provided significantly higher shear strength compared to PUR-bonded wet TCC joints. In wet TCC joints, the average shear strength of epoxy- and PUR-bonded wet TCC joints was reduced by about 46.7% by increasing the waiting time from 0 to 72 min (gelation time). In the case of comparison of dry and wet TCC joints, it was shown that the average shear strength of PUR-bonded dry joints was higher than that of PUR-bonded wet TCC joints. Moreover, a failure in dry TCC joints occurred in the adhesive–concrete surface. Frohnmüller et al. [26] presented new findings on adhesively bonded timber–concrete composites with prefabricated concrete parts. The tests were carried out using new high-viscous polymer mortar as adhesive and an epoxy resin. They observed that polymer mortars showed strong advantages in terms of applicability and bridging of gaps in comparison to the less viscous epoxy adhesive.

Tannert et al. [27] noticed that hybrid shear connectors formed by the combination of mechanical fasteners (self-taping screws) with adhesive bonding can provide high stiffness under service loads (Serviceability Limit State; SLS) and high ductility at the level of failure loads (Ultimate Limit State; ULS). Furthermore, they showed that results obtained on small-scale samples were representative for full-size structures. It has been proven that the design of hybrid structures is possible with a relatively simple γ-method.

The use of analytical methods to solve adhesive joint problems becomes difficult as the complexity of the joints increases, so it is necessary to use numerical methods such as Finite Element Modelling (FEM) to correctly predict the behavior of adhesive joints. The finite element (FE) method, the Boundary Element (BE) method, and the Finite Difference (FD) method are the major numerical methods for solving adhesive joint problems, but the most commonly used method in context of adhesively bonded joints is the FEM [28]. Tannert et al. [29] examined the influence of the bond length on the strength of adhesively bonded hardwood joints using a 2D eight-node orthotropic element model. Frohnmüller et al. [26] used 2D FE for the calculation of the deformations and stresses of Adhesive Timber Concrete Composite (ATCC) beams. Sebera et al. [30] developed a numerical finite element model applicable for analysis of fracture problems in mode II. The FE models included 2D geometry of the bond line and cohesive law fitted on the outputs of the experimental measurement. The 2D FE models showed the influence of the friction coefficient on the outputs of the three-point end-notched flexure test. Based on the 3D model, it was noticed that to avoid plastic deformation below the load head, it is necessary to have a span-to-height ratio greater than 17.

A widely utilized approach for representing and simulating the initiation and propagation of cracks and debonding in composite materials is the Cohesive Zone Model (CZM). This approach is based on the definition of a traction–separation law, which governs the relationship between stresses and displacements at the adhesive interface. In other words, it considers the deterioration of material properties within the damage zone [31]. The behavior at the contact interface is characterized by an initial elastic response, followed by damage initiation and progressive degradation once a critical traction or displacement is reached. CZMs have been extensively validated in studies dealing with fracture mechanics, particularly in bonded composite materials and interfaces between dissimilar materials [32,33]. The versatility of CZMs lies in their ability to model the gradual failure of adhesive joints without requiring an explicit representation of the crack path, making them ideal for applications in composite structures, brittle materials, foams, and other multi-material systems [34,35,36]. For instance, in a recent study, CZMs were employed to model the response of highly flexible adhesives across a broad range of strain rates and temperatures. By calibrating the CZM for specific reference conditions, accurate load–displacement predictions were achieved, even for complex failure mechanisms [37].

It should be emphasized that most of the previous works describe mechanical or hybrid connections, while in the case of adhesive connections, there is a gap; therefore, further research is needed to expand the knowledge. Furthermore, it should be clearly highlighted that no prior studies of this nature have been conducted to date. The unique aspect of this research lies in the use of prefabricated glue joints composed of flexible polymer adhesives within TCC structures, arranged in various configurations. Consequently, the results of this study cannot be directly compared to any previous research. The presented research on flexible systems is an original idea. The experimental campaign included various bonding techniques, which is important in terms of the workability of the real structures. The tested flexible joints enable transfer of high loads and significant deformations with increased ductility and can replace standard mechanical connections in timber structures.

The successful implementation of adhesive bonding in real TCC floor structures remains a challenging task. This difficulty is particularly pronounced when using flexible adhesives, which offer a better stress distribution across the joint but suffer from the limitation of a shorter open/bond time, making them less practical for large-scale applications. One potential solution to this problem is the use of prefabricated flexible adhesives.

Considering the previously described state of the art, a research program has been developed focusing on the following objectives:

- Optimization of bonding performance and stiffness, taking into account the orientation of the prefabricated fastener in order to identify configurations that increase efficiency while minimizing material consumption;
- Verification and refinement of numerical models that predict stress–slip behavior and use them to analyze the stress distribution in the glue and therefore optimize joint configurations;
- Analysis of failure modes and joint ductility to ensure the proper structural performance of prefabricated adhesive joints in TCC systems.

## 2. Experimental Program

### 2.1. Specimen Description

**Figure 1:**
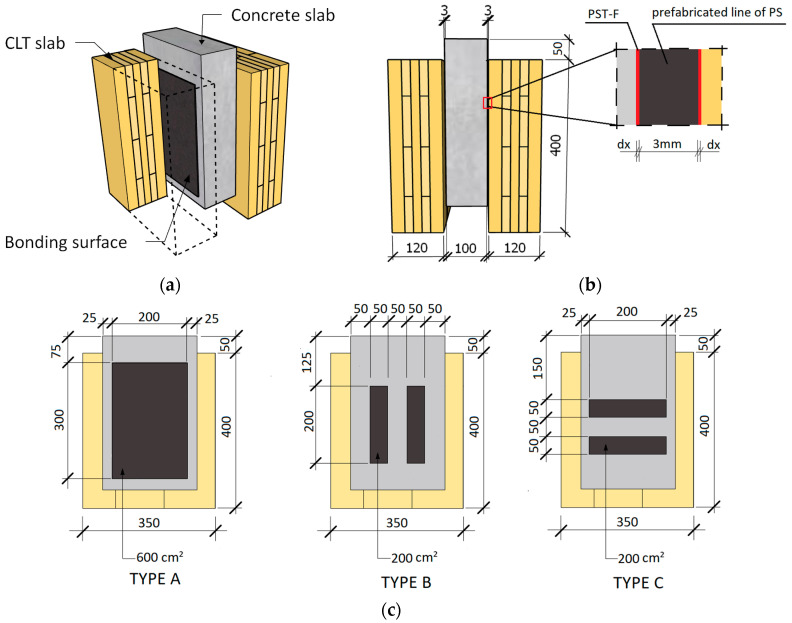
Geometry of the specimens: (**a**) 3D view; (**b**) front view; (**c**) view of the three types of bonding surface.

The experimental program included investigating innovative glued connections via push-out tests followed by the standard procedure provided in EN 26891 [38]. The samples consisted of a central piece of precast concrete block, two lateral CLT elements, and a shear connector made of innovative 3 mm thick prefabricated flexible joints and quick-setting polyurethane F&R PSTF-S, in each shear plane. In order to investigate the influence of the bonding surface and its arrangement in relation to the direction of loading (in various loading situations: cyclic SLS, short-term creep, cyclic ULS, and loading up to failure) on the joint stiffness and slippage, the samples were divided into three types: A, B, and C. Depending on the type, the bonding surfaces were type A = 2 × 60,000 mm^2^; type B = 2 × 20,000 mm^2^ in the form of prefabricated strips parallel to the load axis; and type C = 2 × 20,000 mm^2^ in the form of strips perpendicular to the load axis (see Figure 1c). The dimensions of the lateral elements made of CLT slabs were 400 × 350 × 120 mm^3^, as shown in Figure 1. The dimensions of the concrete block were 400 × 250 × 100 mm^3^. The prefabricated flexible adhesive layers (3 mm thick) were prepared 21 days before bonding in special silicone molds and cut to the required size depending on the type of sample (A = 200 × 300 mm^2^; B = 2 × 50 × 200 mm^2^ and C = 2 × 50 × 200 mm^2^).

**Figure 2:**
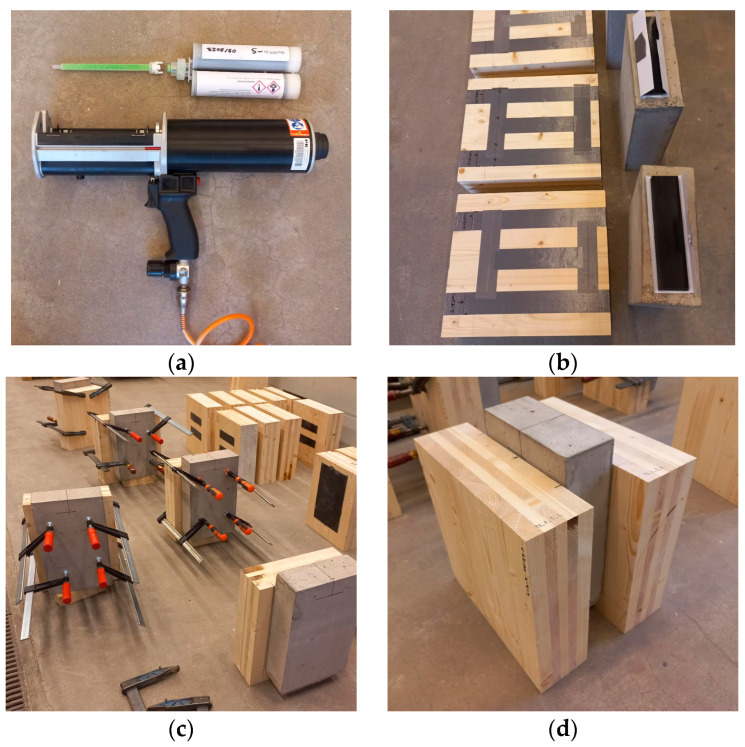
Fabrication process of TC connections: (**a**) PSTF-S view; (**b**) component preparation; (**c**) gluing process; (**d**) view of the completed specimen.

Five replicates were tested for each of the three types of TCC connections, providing sufficient data to analyze the mechanical properties of the joints. As for the fabrication process, glue strips were prefabricated initially and cut to the required size for the different connection types. In the next step, the prefabricated flexible adhesive strips were first glued with a thin layer of quick-setting polyurethane adhesive PSTF-S on the cleaned and dust-free surface of CLT, and then the combined elements were glued on the cleaned and dust-free concrete slab on both sides. Before gluing, the concrete and CLT surfaces were properly protected (see Figure 2b). PSTF-S was applied from 380 mL cartridges using a pneumatic gun and special mixing ejector (see Figure 2a). Screw clamps were used for the geometrical stabilization of the sample. The manufacturing procedure for the TC-B type and TC-C type connection specimens followed the same process as above.

### 2.2. Material Properties

The mechanical characteristics of the CLT elements were supplied by the manufacturer, and they were as follows (Table 1). The average density was 420 kg/m^3^ and moisture content was 12%. The CLT slabs consisted of five layers (30–20–20–20–30 mm) and were made of C24 timber boards made from Norway spruce (*Picea abies* L.).

The prefabricated concrete units were produced in the precast plant from Heidelberg materials in Vislanda, Sweden. The design value of the characteristic compressive strength was f_ck_ = 30 MPa and modulus of elasticity was E_cm_ = 33,000 MPa.

The prefabricated glued connection was made of a two-component polyurethane adhesive with the trade name PS, distributed by the FlexAndRobust System (F&R) company from Cracow, Poland. A fast-setting adhesive with the trade name PST-F (distributed by F&R) was used to connect the prefabricated strips of PS with the remaining elements. The PS is a solvent-free, elastic, two-component, polyurethane-based adhesive able to provide a more uniform stress distribution compared to conventional rigid connectors. In addition, connections using this material are additionally characterized by sound-absorbing properties and vibration damping. The adhesive was mixed as described in the product data sheet. The PST-F is a solvent-free, elastic, two-component, polyurethane-based adhesive applied from 380 mL cartridges using a pneumatic gun and special mixing ejector (see Figure 2a). The basic properties of the adhesives PS and PSTF-S are presented in Table 2. More advanced properties of the presented polyurethanes can be found in [39,40,41,42], where the strain increment speed, impact of elevated temperature, and aging in different corrosive factors was examined.

### 2.3. Test Setup and Loading Protocol

**Figure 3:**
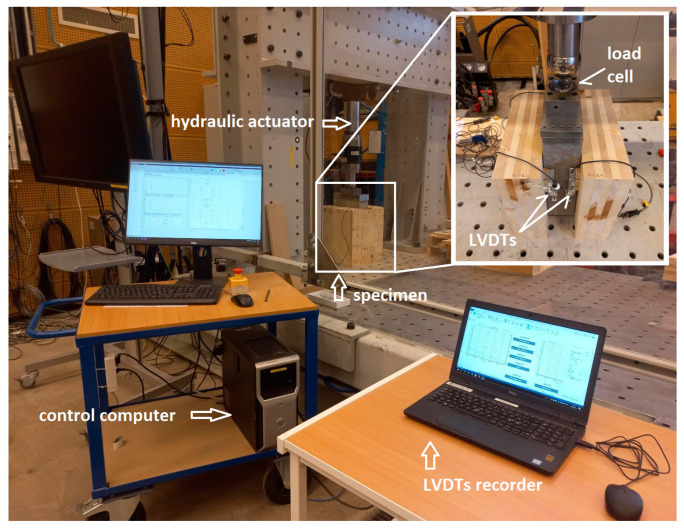
Test setup. Hydraulic testing machine and data acquisition system for the push-out tests.

The push-out tests were performed following EN 26891 [35] with some modifications described in detail in the paragraph below. The specimens were tested on an MTS universal testing machine with a load capacity of up to 300 kN in the laboratory of the Department of Building Technology Laboratory of Linnaeus University in Sweden. The test setup for the push-out test is presented in Figure 3. The load was applied to the top of the concrete element, where a steel plate was positioned for distributing the load from the testing machine. To measure the relative slip between timber and concrete during the test, four Linear Voltage Displacement Transducers (LVDTs) were used, two on each face of the specimen. The relative slip is presented as the average of the four LVDT sensors. To prevent separation and pre-stress forces perpendicular to the shear plane, plywood boards were installed, following the recommendation of restraints from Annex C of CEN/TS 19103:2022 [43] of EC 5.

**Figure 4:**
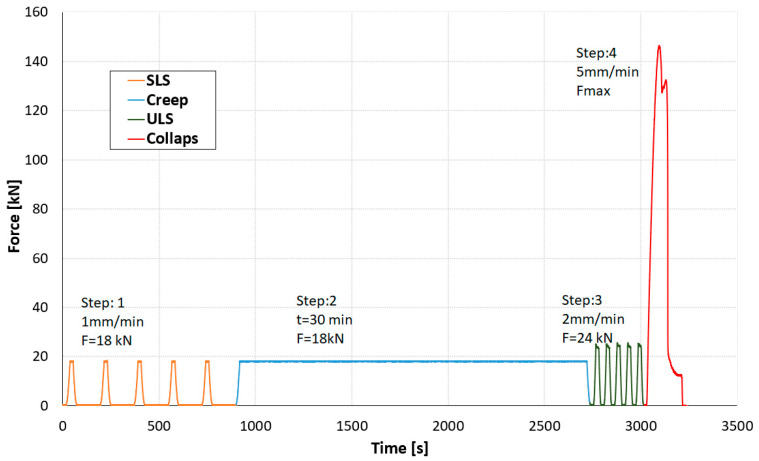
Exemplary loading procedure for a given sample.

According to EN 26891 standard [38], the load should be, at the first loading step, linearly applied up to almost 40% of the estimated failure load ($F_{est}$) within two minutes and maintained for 30 s at this level. The load should then be reduced to 0.1 $F_{est}$ and maintained for 30 s. Thereafter, the load shall be increased until the ultimate load or slip of 15 mm is reached. For the purposes of the experiment, changes were made to the loading protocol in order to obtain more extensive data. The following four main steps were adopted (see Figure 4):

1. The first step, corresponding to the Serviceability Limit State (SLS), consisted of 5 cycles of loading the sample up to 18 kN, with loading and unloading controlled by a displacement rate of 1 mm/min.
2. The second step was the short-term creep investigation on SLS load situation. The specimen was loaded for 30 min with a constant load of 18 kN, with loading and unloading controlled by a displacement rate of 1 mm/min.
3. The third step corresponded to the Ultimate Limit State (ULS), consisting of 5 cycles of loading the sample up to 24 kN, with loading and unloading controlled by a displacement rate of 2 mm/min.
4. The fourth loading step was the loading up to the failure of the connection. The load was increased constantly with a displacement rate of 5 mm/min until the failure of the connection occurred.

The load levels (18 kN and 24 kN) were calculated for the reference glued connection (type A = 2 × 60,000 mm^2^) assuming the real TCC floor solution (consisting of a CLT slab and concrete slab) as presented in [44]. It was assumed that the ceiling with dimensions of 6.50 × 2.34 m was simply supported and loaded with a uniformly distributed load. The self-weight of the ceiling was assumed as $g_{k}$ = 5.20 kN/m^2^, and the value of the imposed load established as $q_{k}$ = 2.5 kN/m^2^ according to [45]. The load combination was assumed according to equation 6.10 for SLS and 6.10b for ULS from the Swedish national annex of EC 0 [46]. For the above assumptions, the maximum shear force at the supports was determined for the two design situations using

(1) $$V_{SLS}=\frac{q_{SLS}\cdot l\cdot a}{2}$$

(2) $$V_{ULS}=\frac{q_{ULS}\cdot l\cdot a}{2}$$

where ${q_{SLS}=7.70\;\frac{kN}{m^{2}},q}_{ULS}=9.95\;kN/m^{2},a=1\;m,l=6.5\;m.$

The value of shear stresses in the glued connection between the CLT slab and the concrete slab for SLS and ULS was determined using the formula

(3) $$\tau_{\left(x,z\right)}=\frac{V_{SLS/ULS}}{{EI}_{ef}\cdot b}\left[\sum_{i=1}^{n-1}E_{i}bh_{i}a_{i}+E_{n}b(z-\sum_{i=1}^{n-1}h_{i})\cdot (z_{NA}-z+\frac{z-\sum_{i=1}^{n-1}h_{i}}{2})\right]$$

where $V_{SLS/ULS}$ is the shear force for SLS and ULS design situation, respectively; ${EI}_{ef}$ represent the effective bending stiffness; b  = 1 m, $h_{i}$ is the height of each layer, z is the z-coordinate where the shear stress is of interest, $z_{NA}$ is the z-coordinate of the neutral axis, and n represents the number of the layer where the shear stress is of interest.

Knowing the shear stress value, the shear force values for SLS and ULS were calculated for the reference bonding surface ($A_{type\;A\;}=60,000\;mm^{2})$ using the formulas

(4) $$F_{SLS}=2\cdot \tau_{\left(x,z\right)SLS}\cdot A_{type\;A\;}=18.3\;kN$$

(5) $$F_{ULS}=2\cdot \tau_{\left(x,z\right)ULS}\cdot A_{type\;A\;}=23.6\;kN$$

Ultimately, slightly lower force values were adopted in the experiment: 18 kN and 24 kN for SLS and ULS, respectively. The same load values were used for the remaining sample types B and C.

Short-term creep tests (step 2 of the procedure) were performed based on the principles of identifying creep parameters based on short-term creep tests developed for identifying creep properties of concrete [47,48].

## 3. Test Results and Discussion

The current study investigates the influence of the bonding surface area and its orientation to the load direction on the longitudinal shear load-bearing capacity, stiffness, and slip modulus of the connection. In addition, the impact of cyclic loads and the short-term creep behavior were analyzed. The results revealed a nonlinear behavior of flexible adhesives, characterized by advantageous reduction of stress concentration and uniform stress distribution along the bond line (change from brittle failure characteristic for glued joints on flexible ones). Based on the experimental data characteristic parameters, like slip modulus, stiffness, shear stresses, engineering shear strain, yield point, ductility, and creep factor, were identified. In addition, the failure modes were described.

### 3.1. Load-Bearing Capacity and Load–Slip Behavior

Table 3 shows the results, which include maximum loads ($F_{max}$) and relative slips ($\delta_{rel}$) for each type of specimen obtained from step 4 of the loading procedure (loading until the collapse of the connection). The average load-bearing capacity of the type A connection is much higher (126.6 kN) than that of the other specimens due to the bonding surface being three times larger. However, the relationship between the load capacity and the size of the bonding surface is not linear. It can also be seen from Table 3 that changing the orientation of the adhesive strips depending on the load direction from vertical (type B) to horizontal (type C) does not have an impact on the load-bearing capacity of the connection, the obtained difference is only 2.1 kN, i.e., 3%. Interestingly, slightly better results were obtained for the horizontal arrangement (70.2 kN). Moreover, the larger bonding surface (type A) was characterized by a higher coefficient of variation (CoV) than type B and C bonds, where the coefficients of variation were very similar: 15.6 and 15.8%, respectively. The highest average slip was recorded for type B samples, amounting to 2.16 mm and being 4% greater than type C samples and over 14% greater than type A samples. It can be seen that the change in the arrangement of the polyurethane strips relative to the direction of force did not have a large impact on the change in the average slip value. Additionally, the slip for type B samples was characterized by the lowest coefficient of variation, amounting to 9.6%. Table 3 also presents the results for the numerical model described in Section 4.

**Figure 5:**
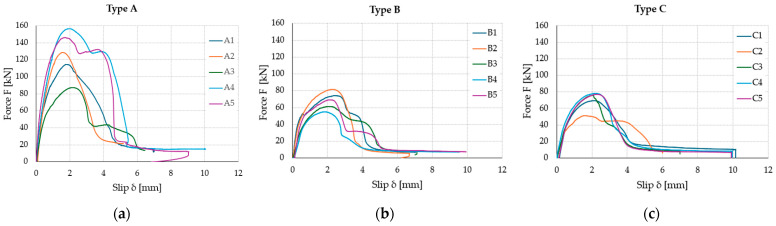
Load–slip curves of the five tested specimens for each connection type: type A (**a**); type B (**b**); type C (**c**).

The tested load–slip curves of the five TCC specimens for each type are depicted in Figure 5. The load capacity of the type A connection increased rapidly at the beginning of the loading process, linearly to a value of approximately 60 kN. The exception was sample A3, where, after removing the CLT boards, the smallest active surface involved in transferring the shear load was observed. The low increase in load capacity was related to the type of damage, i.e., a problem in adhesion between the adhesive and the concrete slab and cohesive failure in the concrete, which led to a smaller load capacity. In this case, only 53.2% (on the right) and 46.7% (on the left) of the bonding surface were involved in load transfer. A similar problem was observed for sample C2. The form of adhesive failure is described in detail in Section 3.4.

### 3.2. Slip Modulus and Stiffness

The slip modulus of the connection was calculated according to EN 26891 [38], but with some simplifications due to the applied loading procedure considering the SLS and ULS load steps of a real TCC slab. The slip modulus $K_{s}$ which is usually used to determine the serviceability stiffness, corresponds to the slope of the load–slip corresponding to the load, ranging from 10% to 40% of the failure load, and is calculated according to the formula

(6) $$K_{s}=\frac{0.4\cdot F_{est}}{v_{i,mod}}$$

(7) $$v_{i,mod}=\frac{4}{3}\;(v_{0.4}-v_{0.1})$$

where $v_{0.1}$ and $v_{0.4}$ represent the displacements recorded under loads of 0.1 $F_{est}$ and 0.4 $F_{est}$.

The ultimate stiffness of connection is defined as the slip modulus $K_{u}$. According to the EN26891 standard [38], the slip modulus for the ultimate limit state can be calculated as

(8) $$K_{u}=\frac{2}{3}\cdot K_{s}$$

The above rules were applied to the last step of the experiment, i.e., increasing the load to collapse. However, in this test, the $F_{est}$ was replaced by the maximum load capacity $F_{max}$ that was obtained from the experiment. The stiffness of the joints k, i.e., the smeared slip modulus was determined by dividing the slip modulus with the total estimated working glued area $A_{W}$ for both joints:

(9) $$k=\frac{K}{A_{W}}$$

Table 4 shows the results, which include the last step of the loading protocol. The results for slip modulus were calculated with Equations (8) and (9). The average value of slip modulus for the SLS $\left(K_{s}\right)$ of the type A connection is 34.8% and 47.8% greater than the slip modulus $K_{s}$ for samples B and C, respectively. It can also be seen from Table 4 that changing the orientation of the adhesive strips, depending on the load direction from vertical (for type B) to horizontal (type C), causes a decrease in the slip modulus from 115.5 kN/mm to 92.5 kN/mm, which gives a reduction of 19.9%. Additionally, the slip modulus for type C samples was characterized by the lowest coefficient of variation, amounting to 13.3%. The stiffness of the joints (smeared slip modulus) was calculated with Equation (9). The working surface of the adhesive $A_{W}$ was estimated based on the analysis of photos carried out in the ImageJ program. A detailed summary of the surfaces, divided into left and right sides of the connection, is presented below in Table 5.

### 3.3. Failure Modes

**Figure 6:**
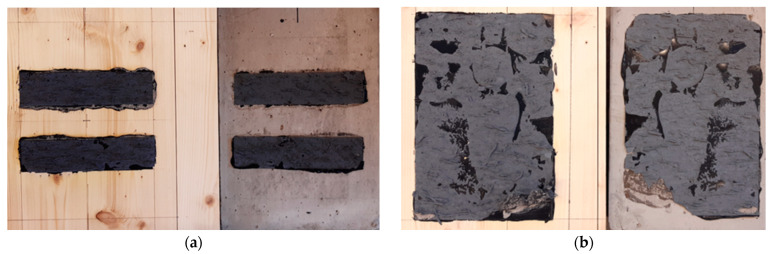

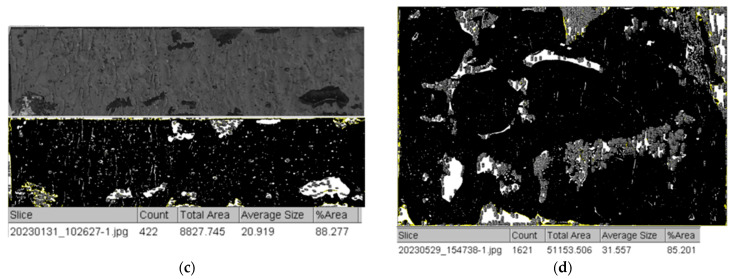
Failures of TC-connections: (**a**) cohesive failure in the polymer in the sample type C-1; (**b**) mixed failure: adhesive failure at the concrete–polymer interface (ac-p) with cohesive failure in the polymer (cp) in the type A sample; (**c**) bonding surface for the selected sample type B-1; (**d**) bonding surface for the selected sample type A-4.

The failure modes were compared and described based on EN ISO 10365:2022 [49]. To better identify damage patterns, extended marking was proposed, taking into account the materials used in connection (timber and concrete). The description was divided into left and right bond sides using the following designations: cohesive failure in timber (ct), cohesive failure in the polymer (cp), cohesive failure in concrete (cc), adhesive failure at the timber-polymer interface (at-p), adhesive failure at the concrete–polymer interface (ac-p), and adhesive failure at the polymer-polymer interface (ap-p). To further visualize and investigate the failure modes of the TC connections, the concrete block was removed. Initial deformations were, as expected, a result of elastic shear deformations of the adhesive due to higher flexibility of the adhesive in comparison with CLT or concrete slab. The failures of TC connections in most cases were due to polymer cohesive failure or problems with polymer-to-polymer adhesion or polymer-to-concrete adhesion (see Figure 6). It should be added that the destruction of the polymer was an expected form of damage; the remaining forms that occurred are the result of application challenges.

The estimation of the working glued area $A_{W}$ was made in the program ImageJ (version 1.54g). Both sides (left and right) of the joint of all samples were photographed and then imported into ImageJ, where it was possible to scale the images to the desired area of interest and have it calculated by the software. The estimated glued area was then used for determination of the nominal stress with respect to the two joints. The sum of the left and right connections shown in Table 5 was used as $A_{W}$. It can be observed that for the smaller gluing surfaces of types B and C, the average utilization is at a much higher level, within the range of 81–84%, respectively. In comparison, for type A connections, the average utilization is at the level of 57%. The small active surface of the connection resulted in low load-bearing capacity. For sample A3, where the lowest maximum force was recorded ($F_{max}$ = 87.0 kN), the use of the bonding surface was only 60,094.9 mm^2^, which is 50.1% of the entire bonding surface.

### 3.4. Shear Stresses and Engineering Shear Strain

The shear stresses (engineering stress) were calculated considering the effective estimated working glued area $A_{W}$ as

(10) $$\tau =\frac{F_{max}}{A_{W}}$$

**Figure 7:**
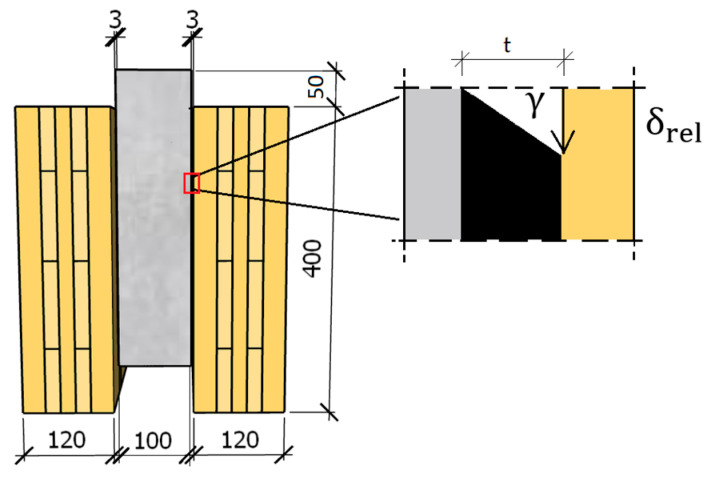
Illustration of the deformed adhesive layer and its shear angle.

Due to the significant thickness of the adhesive, t = 3 mm, large shear deformations were expected. Therefore, the engineering shear strain was calculated according to the formula

(11) $$\varepsilon_{eng}=\frac{\gamma }{\sqrt{1+\frac{\gamma^{2}}{4}}}$$

where γ is the tangent of the angle between the thickness of the adhesive and the relative slip (strain for small deformation); see Figure 7.

**Figure 8:**
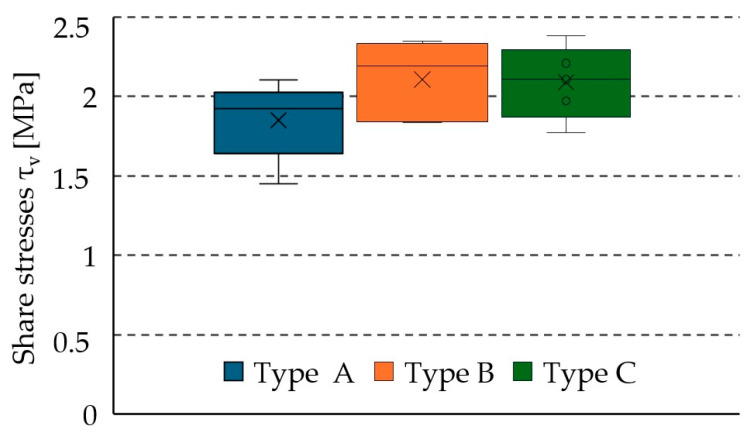
Shear stress results.

Table 6 presents the shear stress results for each of the five specimens of each connection type. The shear stress values do not differ significantly, showing minimal variation, particularly between types B and C, where the difference is only 0.5%. For type A, the average shear stress is 12% lower than for type B. All results exhibit a low coefficient of variation, remaining below 12% (see Figure 8).

### 3.5. Yield Load and the Ductility

**Figure 9:**
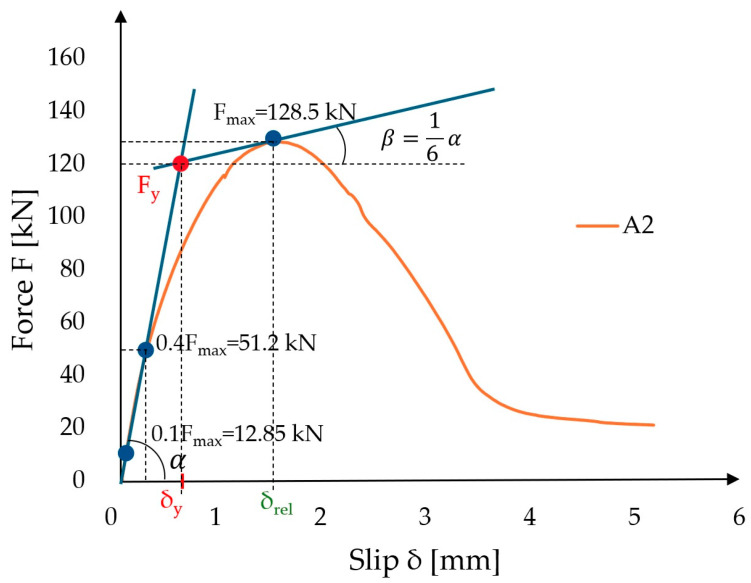
Methods of yield point determination for selected sample, type A, number 2.

The tested load–slip curves of TCC connections do not have a clear yield platform; therefore, it is difficult to determine the yield point. According to the European Committee of Standardization (CEN) 12512:2002 [50] when the load–slip curve does not present two well defined linear parts, the yield values are determined by the intersection of the following two lines: the first line will be determined as that drawn through the point on the load slip curve corresponding to 0.1 $F_{max}$ and the point on the load–slip curve corresponding to 0.4 $F_{max}$; the second line is the tangent, having an inclination of one-sixth of the first line, and runs through the max load point (see Figure 9).

To estimate the ductility of the joints, the yield point must be accurately determined. Ductility can be measured by the ratio between ultimate slip and yield slip:

(12) $$D=\frac{\delta_{rel}}{\delta_{y}}$$

As can be seen from Table 7, the ductility coefficient obtained by Equation (12) ranges from 2.19 to 5.9, indicating that prefabricated adhesive connections can show considerable ductility. The average yield forces $F_{y}$ for the three types of connections A, B, and C were calculated as 119.6 kN, 59.4 kN, and 64.1 kN, respectively, with coefficients of variation (CoV) of 23.4%, 15.2%, and 15.6%, respectively. The yield load, as expected, had the highest value for type A connections, which were 51.1% and 46.4% higher, respectively, compared to type B and C connections. The best fit of the yield force $F_{y}$ between the numerical model was obtained for C-type connections.

### 3.6. The Impact of Cyclic Loads

**Figure 10:**
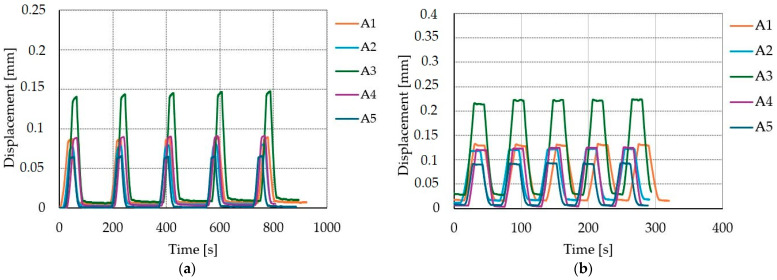
Displacement–time curves for connection type A: (**a**) five cycles of serviceability limit state with a constant load of 18 kN; (**b**) five cycles of ultimate limit state with a constant load of 24 kN.

**Figure 11:**
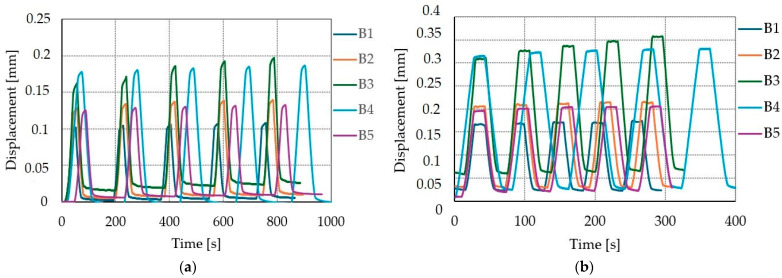
Displacement–time curves for connection type B: (**a**) five cycles of serviceability limit state with a constant load of 18 kN; (**b**) five cycles of ultimate limit state with a constant load of 24 kN.

**Figure 12:**
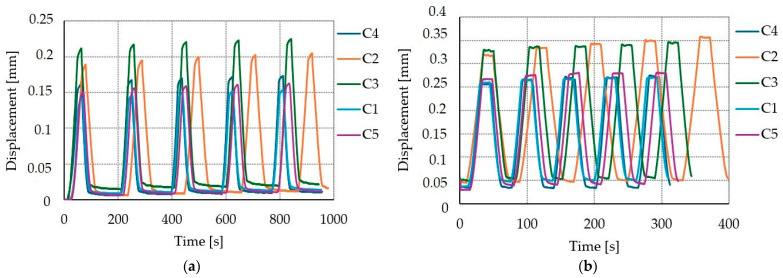
Displacement–time curves for connection type C: (**a**) five cycles of serviceability limit state with a constant load of 18 kN; (**b**) five cycles of ultimate limit state with a constant load of 24 kN.

The impact of cyclic loads on displacement is shown on Figure 10, Figure 11 and Figure 12. The average increase of displacement from first to fifth loading cycle for SLS were 5.1%, 9.5%, and 7.2% for A, B, and C connections, respectively. When comparing the ULS and the SLS displacement–time curves (see Figure 10, Figure 11 and Figure 12), it is possible to see that the displacement is higher in the ULS load case, which is reasonable since the load application is higher for ULS than SLS. The average increases in displacement from the first to fifth loading cycle in the ULS were 4.9%, 6%, and 6.2% for A, B, and C connections, respectively.

### 3.7. Creep Impact

**Figure 13:**
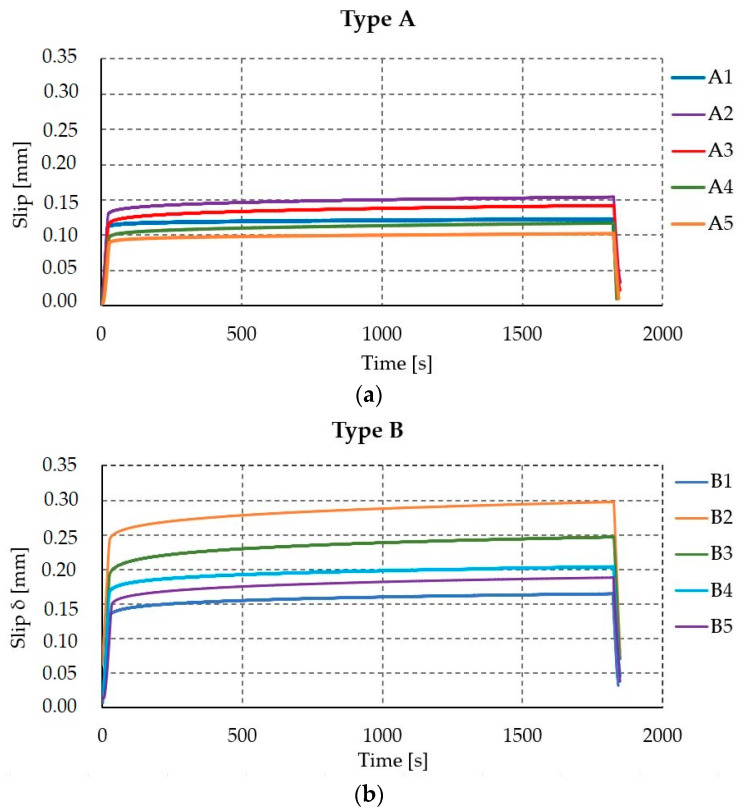

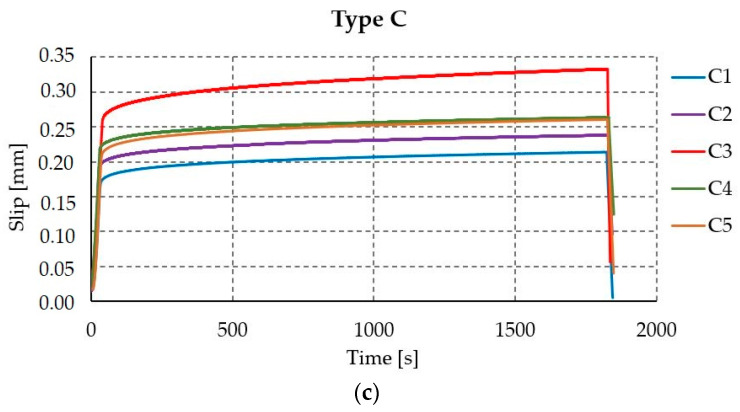
Displacement–time curves, 30 min of creep investigation with a constant load of 18 kN: connection type A (**a**); connection type B (**b**); connection type C (**c**).

The displacement–time creep diagram (see Figure 13) shows clearly that the displacement increased on all of the specimens over time. All involved materials are creep-active, but due to the stiffness ratios, the main contribution in the experiments is coming from the adhesive (due to the viscous characteristics of the adhesive). The average increase in displacement after 30 min of constant loading was 20.4%, 29.0%, and 26.3% for types A, B, and C, respectively (see Table 8). It should be added that the increase in displacement is measured from achieving instantaneous displacement at a force of 18 kN. A more detailed description of the creep effect for the presented samples is presented in paper [51].

The deformation caused by creep is often described by the creep coefficient, which can be defined as

(13) $$\varphi =\frac{\delta_{t}-\delta_{inst}}{\delta_{inst}}$$

where $\delta_{t}$ is the relative slip of the joint at time t and $\delta_{inst}$ denotes the instantaneous slip (elastic) at a force of 18 kN.

**Figure 14:**
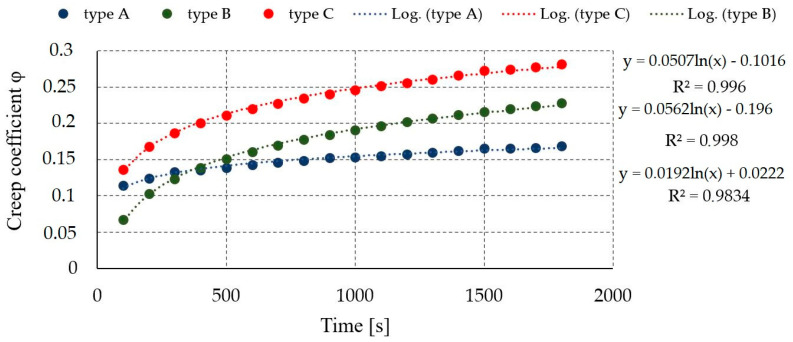
Experimental creep coefficient of TCC.

The average values of creep coefficients of TCC joints determined for points with a time step of 100 s for samples type A, B, and C are shown in Figure 14.

## 4. Numerical Model

The introduction of a Finite Element (FE) model is necessary to extend the case study and to investigate the variability of the adhesive mechanical parameters, like tangential stiffness, that otherwise would be impractical to be analyzed just with laboratory tests.

### 4.1. Model Description

A Finite Element numerical model that simulates in a numerical environment the shear-slip test design is built in Abaqus/CAE. The model represents the two panels of CLT and the concrete block. Regarding the mechanical parameters (Young’s Modulus, Poisson ratio) of concrete and timber, the values in the model are the same that are reported in Table 1 and Table 2 in Section 2.

**Figure 15:**
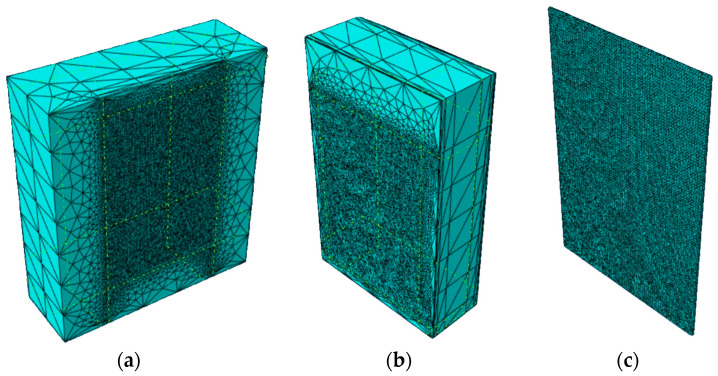
CLT element with adaptive mesh (**a**); concrete element with adaptive mesh (**b**); polyurethane layer with 3 mm mesh (**c**).

Several modeling approaches were initially evaluated to properly represent the adhesive layer behavior. The first attempt involved using an adaptive mesh that was refined only in correspondence with the adhesive layer, with a minimum step size of 3 mm, to match the polyurethane layer thickness, as can be seen in Figure 15. However, this solution proved to be computationally unfeasible, as the fine mesh exponentially increased the number of finite elements, leading to prohibitive processing times. Attempts to optimize the solution by modifying the mesh step size and interface interaction parameters were unsuccessful, as the various Abaqus integration methods consistently failed to handle the large displacements observed in the test (resulting in non-convergent solutions for most interface stiffness values).

**Figure 16:**
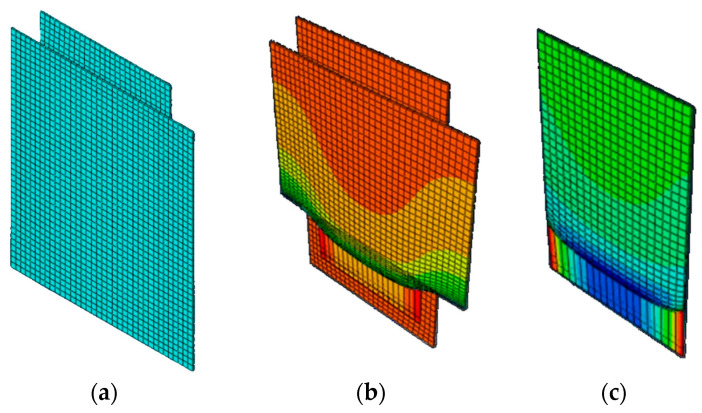
Reduced model mesh: (**a**) deformation reached in logarithmic scale with CLT, concrete and polyurethane (**b**) deformation reached in logarithmic scale of polyurethane layer (**c**).

A reduced model was also investigated, where only 3 mm thick elements were considered to maintain comparable thicknesses, with boundary conditions replicating the actual dimensions of the wooden and concrete elements. The representation of the model is reported in Figure 16. However, this approach also failed to achieve convergence due to the high deformations that the elements corresponding to the adhesive layer needed to sustain.

The key challenge lies in the significant scale difference between the adhesive thickness and the experimentally observed displacements, combined with the hyperplastic (non-linear) nature of the polyurethane. In large displacement regimes, the stiffness matrix becomes strongly dependent on displacement evolution over time, requiring the mechanical problem to be solved by applying the Principle of Virtual Work to n deformed (non-linear) configurations. Additionally, when using the Newton–Raphson method, the software must reconstruct matrices at each iteration, which for highly distorted elements are typically ill-conditioned, making the iterative process highly unstable. Alternative attempts using non-linear analysis methods like the central difference method, while not requiring convergence, yielded highly unstable solutions, consistent with the Courant–Friedrichs–Lewy stability condition, requiring sufficiently small time steps compared to the system’s natural frequency.

Due to the small thickness of the adhesive layer, the polyurethane is not directly modeled in order to avoid the typical convergence issues that can emerge when analyzing systems that include components of very small size in comparison to the overall system. Instead, a Cohesive Zone Model (CZM) is employed to represent the adhesive characteristics at the contact areas between the CLT and concrete elements.

The CLT and concrete blocks were modeled as 3D solid structures using eight-node brick elements (C3D8R) with reduced integration. The polyurethane adhesive layer between the blocks was instead represented by means of a cohesive surface interaction which introduces nonlinear deformation up to failure. The surface interactions simulate the mechanical cohesive behavior of the blocks and control their relative slip under the applied load. The cohesive criterion simulates the adhesive response to loading, and, in combination with a damage criterion, it allows the simulation of even the initiation and evolution of damage and the failure of the joints. The Maximum Principal Stress (MPS) is chosen as the damage criterion since the nature of the physical experiment allowed a better control over measurements of the applied load, rather than relative sliding between the blocks.

The damage evolution is defined by a displacement-based softening model that simulates the gradual loss of stiffness in the adhesive joint once the stress in a certain direction exceeds the threshold in that direction. The softening law that governs the reduction in load-bearing capacity as the relative displacement increases is defined based on the experimental data obtained during the laboratory test.

### 4.2. Model Validation and Numerical Analysis

A dynamic explicit analysis is conducted to simulate the shear test, where the interaction between the blocks is subjected to controlled displacements. Bulk viscosity was introduced to stabilize the model during the dynamic simulation.

The methodology employed in explicit dynamics analysis are based on the formulation of an explicit integration rule and the deployment of diagonal mass matrices. The motion equations of the generic element are calculated through the implementation of an explicit central-difference integration procedure [52].

The definition of the characteristic parameters that describe the behavior of the adhesive interfaces is accomplished through a tuning process developed on the basis of the available experimental data.

**Figure 17:**
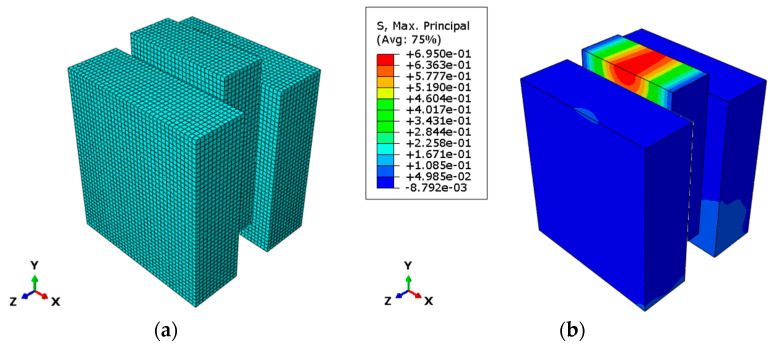
Model of the Type A specimen: (**a**) mesh; (**b**) trend of stresses.

Figure 17 shows the global model of the Type A specimen with the mesh and presents the trend of stresses within the different elements.

**Figure 18:**
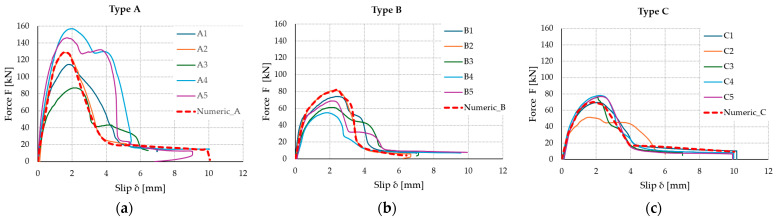
Load–slip curves including numerical investigation: connection type A (**a**); connection type B (**b**); connection type C (**c**).

The FE model is then validated by a comparison of the numerical results with the experimental ones. This comparison shows that the different numerical models are able to simulate the real behavior of the mechanical system with a good degree of approximation. In Figure 18a–c, the comparison in terms of force–slip diagrams is presented.

As can be seen in Figure 18a–c, the numerical model always succeeds in simulating the softening of the polyurethane joint. In the case of specimens A and C, the numerical curve is closer to the average values defined on the different experimental tests. Only in case B did the simulation return slightly higher values; however, they were very close to those of the experimental case that showed higher strength (B2). This effect is due to the fact that, in the case of the type B sample, it is not possible to further reduce the values of the tangential stiffness τ_ss_ and τ_tt_, as even small reductions result in strong instabilities in terms of solution stability.

Conversely, the parameters that govern the initiation of the joint damage phenomenon were found to be general and valid for each specimen. Instead, a softening evolution law was defined for each test type. For each sample, the damage trend was described by a function (defined in discrete form using tables) that correlates a damage variable (varying between 0 and 1) with plastic strain. The reason is based on the fact that when damage occurs in a material, the stress–strain relationship is no longer an accurate representation of the material’s behavior. The continued utilization of the stress–strain relation results in a significant dependency on the mesh based on the strain location. The calibrated parameters that define the interaction properties for the type A sample are reported in Table 9 and Table 10.

The dynamic analysis conducted in a numerical environment is extremely useful because of its capacity to monitor several quantities that otherwise could be difficult to measure during laboratory tests. In fact, in this work, the following parameters have been observed:

- CSLIP1 and CSLIP2, which represent the mutual sliding between master nodes and slave nodes of the contacting surfaces;
- CSLIPEQ, which is the total sliding length of the nodes;
- CSMAXCRT, which is the damage degree reached by the contact surfaces.

**Figure 19:**
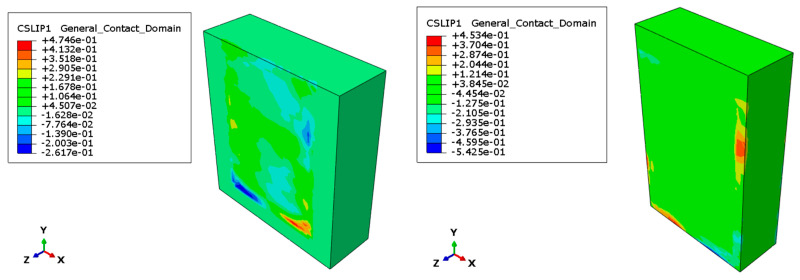

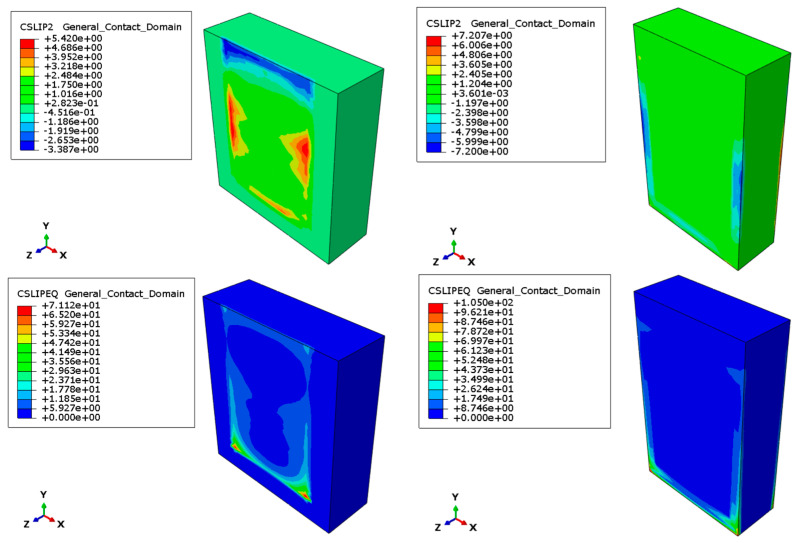
Trends for CSLIP.

In the Figure 19, the results obtained are graphically represented by diagrams that show the values of the aforementioned parameters, at the end of the analysis. For the sake of brevity, only the results for the type A specimen are reported.

Another important aspect that could be emphasized through numerical analysis is the evolution of polyurethane joint damage. In fact, as mentioned earlier, a damage function can be defined in the model that defines the evolution of the softening behavior of the material.

**Figure 20:**
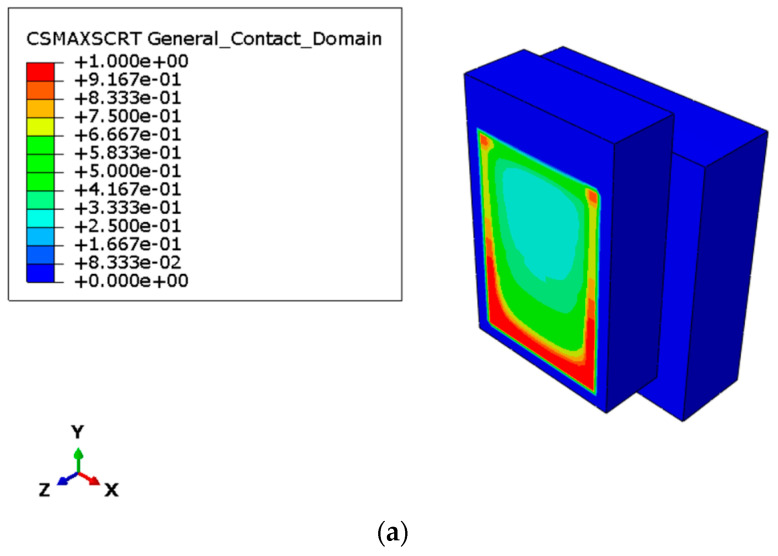

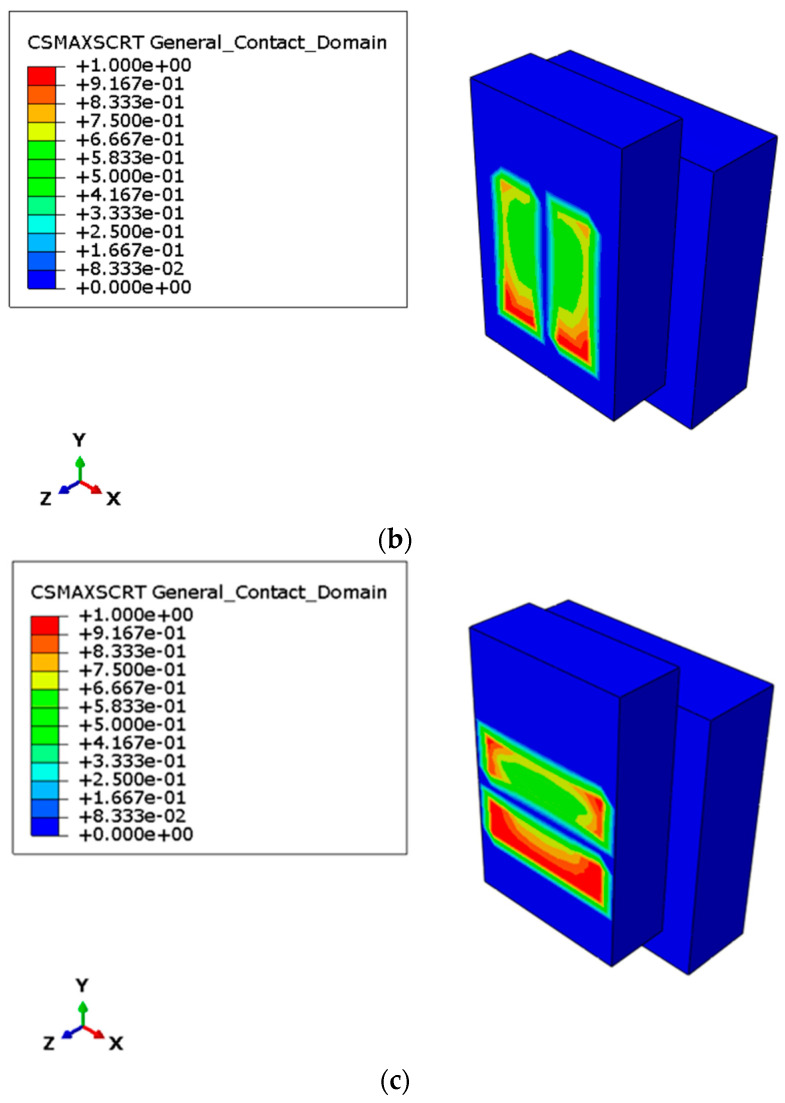
Numerical simulation—damage variable at the time: (**a**) connection type A; (**b**) connection type B; (**c**) connection type C.

It is notable that the numerical model demonstrated a high level of correspondence with the laboratory tests, even in the representation of the detachment regions. Indeed, Figure 20a–c show diagrams for the damage variable at the time when joint damage is initiated, and concrete block slip is activated. As can be seen, especially for the case of specimen C, the damage trend clearly resumes that observed experimentally. The same phenomenon is less evident in the case of specimen A, probably due to the extension of the cohesive zone and the assumptions of absolute isotropy of the connection.

## 5. Conclusions

Experimental and numerical studies have been conducted to assess the effectiveness of bonding with prefabricated flexible polymer adhesives in TCC structures. In these studies, three different configurations of innovative bonding elements were tested using push-out tests to evaluate their performance. The main findings are summarized as follows:

- The shear resistance of a prefabricated adhesive joint does not grow directly proportionally to the bonding area—a threefold increase in bonding area led to only an 80% increase in failure load. This points to the validity of using more efficient adhesive joints with smaller surface areas, which can help minimize material consumption.
- The initial stiffness of the tested joints is dependent on the surface area of the bonding, as well as the orientation of the prefabricated adhesive fasteners. The stiffness of the joint is approximately 20% greater when the bonding elements are aligned parallel to the load direction (as opposed to perpendicular direction).
- The force–slip curves generated by the numerical model closely matched the experimental results, indicating that the proposed FE model is capable of accurately predicting the full-scale stress–slip response of flexible adhesive joints between timber and concrete. The presented computational model also enables the study of the real stress distribution in prefabricated adhesive joints with different configurations.
- The failure modes of the specimens were mainly characterized by (i) polymer cohesive failure, (ii) problems with polymer-to-polymer adhesion, or (iii) polymer-to-concrete adhesion. The similar magnitudes of the failure loads for these three failure modes suggest that the implementation of these innovative joints was relatively successful.
- The joint ductility coefficient, calculated using the CEN method, ranges from 2.2 to 5.9. This indicates that the new prefabricated joints can exhibit significant ductility, which is the primary objective of replacing rigid epoxy adhesives with flexible polymer joints.

Further research on improving bonding capacity by modifying the concrete surface or enhancing the concrete–adhesive bonding capacity with different types of primers would be the next step. In addition, large-scale tests would presumably show the positive influence of increased bonding thickness and bonding surface through a reduction in the negative effect of stress concentration at the edges or debonding. There are currently no standard methods available for large-scale tests of thick flexible adhesive in timber concrete connections; therefore, further studies are needed in this aspect. In terms of further numerical investigations, the next step would be to focus on including fracture phenomena at the interface between elements and adhesive.

## Figures and Tables

**Table 1:** Mechanical parameters of timber layers used to produce CLT panels.

Material	Young’s Modulus [MPa]	Shear Modulus [MPa]	Poisson Ratio [-]
Norway spruce, strength class C24	E_L_ = 11,000	G_LR_ = 690	ν_LR_ = 0.05
	E_R_ = 700	G_LT_ = 600	ν_LT_ = 0.04
	E_T_ = 400	G_RT_ = 70	ν_RT_ = 0.30

L, R, and T represent the three main directions of wood: longitudinal, tangential, and radial, respectively.

**Table 2:** Properties of adhesives.

Material	Young’s Modulus [MPa]	Tensile Strength [MPa]	Poisson Ratio [-]	Density [g/cm^3^]
PS	20	2.6	ν = 0.47 [39]	1.40
PSTF-S	150	17.3	ν = 0.4 [39]	1.22

**Table 3:** Test results for the investigated connection types A, B, and C: results of the individual specimens—maximum load $F_{max}$ and relative slip $\delta_{rel}$ (corresponding displacement), average value for each connection type of $F_{max}$ and $\delta_{rel}$ and the coefficient of variation (CoV), and the modelling results (FEM) for the average values of each connection type.

Specimen	Type A		Type B		Type C	
	$F_{max}$ (kN)	$\delta_{rel}$ (mm)	$F_{max}$ (kN)	$\delta_{rel}$ (mm)	$F_{max}$ (kN)	$\delta_{rel}$ (mm)
1	114.7	1.81	74.1	2.36	69.5	2.15
2	128.5	1.59	81.7	2.30	51.3	1.60
3	87.0	2.18	61.0	2.19	75.7	2.07
4	156.7	1.95	54.8	1.83	77.9	2.23
5	146.3	1.72	68.8	2.12	76.8	2.32
Average	126.6	1.85	68.1	2.16	70.2	2.08
CoV (%)	21.7	12.2	15.6	9.6	15.8	13.5
FEM	129.3	1.58	82.0	2.47	70.1	1.83

**Table 4:** Test results: slip modulus and stiffness (loading until the collapse).

Specimen	Type A					
	$0.4F_{max}$ (kN)	$v_{0.1}$ (mm)	$v_{0.4}$ (mm)	$K_{s}$ (kN/mm)	$K_{u}$ (kN/mm)	k (N/mm/mm^2^)
1	45.9	0.056	0.266	164	109.3	2.79
2	51.4	0.151	0.353	190.6	127.1	2.85
3	34.8	0.076	0.286	124.1	82.8	2.07
4	62.7	0.077	0.361	166	110.7	2.22
5	58.5	0.044	0.227	240.8	160.5	3.01
Average	50.66	0.08	0.3	177.1	118.1	2.59
CoV (%)	21.7	51.3	19.3	24.2	24.2	16.1
FEM-A	51.7	0.129	0.368	170.5	113.7	-
**Specimen**	**Type B**					
	$0.4F_{max}$ **(kN)**	$v_{0.1}$ **(mm)**	$v_{0.4}$ **(mm)**	$K_{s}$ **(kN/mm)**	$K_{u}$ **(kN/mm)**	k **(N/mm/mm^2^)**
1	29.6	0.048	0.184	162.4	108.3	4.45
2	32.7	0.071	0.26	129.2	86.2	3.71
3	24.4	0.112	0.335	82	54.7	2.46
4	21.9	0.159	0.377	75.2	50.1	2.52
5	27.6	0.164	0.325	128.5	85.7	4.09
Average	27.23	0.11	0.3	115.5	77	3.45
CoV (%)	15.5	46.7	25.4	31.5	31.5	26.3
FEM-B	32.8	0.076	0.263	127.8	85.2	-
**Specimen**	**Type C**					
	$0.4F_{max}$ **(kN)**	$v_{0.1}$ **(mm)**	$v_{0.4}$ **(mm)**	$K_{s}$ **(kN/mm)**	$K_{u}$ **(kN/mm)**	k **(N/mm/mm^2^)**
1	27.8	0.199	0.394	106.4	70.9	3.01
2	20.5	0.083	0.286	75.7	50.5	2.61
3	30.3	0.118	0.387	84.4	56.2	2.35
4	31.2	0.081	0.318	98.3	65.5	3
5	30.7	0.181	0.417	97.8	65.2	2.81
Average	28.09	0.13	0.36	92.5	61.7	2.76
CoV (%)	15.8	41.6	15.4	13.3	13.3	10.2
FEM-C	28.05	0.082	0.332	90.5	60.3	-

**Table 5:** Test results—failure modes and working gluing area, where ap+c denotes mixed failure at the concrete–polymer interface with cohesive failure in the polymer, ap-p+cp denotes the mixed failure of the polymer-polymer interface with cohesive failure in the polymer, and cp means cohesive failure in the polymer.

Specimen	Type A		Type B		Type C	
	Failure Mode—Line of Bonding					
	Left	Right	Left	Right	Left	Right
1	ac-p+cp	ac-p+cp	cp	cp+ap-p	cp	cp+ac-p
2	ap-p+cp	ac-p+cp	cp	cp+ap-p	cp	cp+cc
3	ac-p+cp	cp	cp+cc	cp+ap-p	cp+ap-p	cp+ac-p
4	cp	ap-p+cp	cp+ap-p	cp+ ap-p	cp	cp+ap-p
5	ap-p+cp	ac-p+cp	cp	cp+ ap-p	cp	cp+ap-p
	$\mathrm{Estimated}\;\mathrm{working}\;\mathrm{gluing}\;\mathrm{area}\;A_{W}$ [mm^2^]					
	left	right	left	right	left	right
1	25,025.5	33,790.5	18,067.4	13,922.2	19,179.0	16,113.9
2	31,261.7	35,643.8	19,266.0	15,582.0	12,836.4	16,106.0
3	28,178.9	31,915.9	18,370.5	14,856.6	16,717.2	19,219.8
4	23,455.1	51,153.5	16,784.6	12,983.0	17,759.8	14,965.3
5	52,274.0	27,705.8	18,338.8	13,124.7	18,317.6	16,470.7

**Table 6:** Test results: shear stresses $\tau_{max}$ and engineering shear strain $\varepsilon_{eng}$ of individual tests and average values and coefficient of variation (CoV) of the investigated connection types A, B, and C.

Specimen	Type A		Type B		Type C	
	$\tau_{max}$ (MPa)	$\varepsilon_{eng}$	$\tau_{max}$ (MPa)	$\varepsilon_{eng}$	$\tau_{max}$ (MPa)	$\varepsilon_{eng}$
1	1.95	0.58	2.31	0.73	1.97	0.67
2	1.92	0.51	2.34	0.72	1.77	0.52
3	1.45	0.68	1.83	0.69	2.10	0.65
4	2.10	0.68	1.84	0.58	2.38	0.70
5	1.83	0.61	2.19	0.67	2.21	0.72
Average	1.85	0.59	2.10	0.68	2.09	0.65
CoV (%)	13.3	11.1	11.9	8.6	11.1	12.3

**Table 7:** Test results: yield force $F_{y}$, yield point $\delta_{y}$, and ductility coefficient D of individual tests and average values and coefficient of variation (CoV) of the investigated connection types A, B, and C.

Specimen	Type A			Type B			Type C		
	$\delta_{y}$ (mm)	D (-)	$F_{y}$ (kN)	$\delta_{y}$ (mm)	D (-)	$F_{y}$ (kN)	$\delta_{y}$ (mm)	D (-)	$F_{y}$ (kN)
1	0.60	2.87	107.2	0.36	5.9	59.9	0.73	3.18	63.4
2	0.67	2.37	120.0	0.54	4.2	73.3	0.61	2.63	47.1
3	0.65	3.35	79.9	0.69	3.17	52.8	0.83	2.49	68.3
4	0.89	2.19	151.6	0.77	2.37	50.2	0.71	3.14	71.7
5	0.53	3.24	139.4	0.58	3.66	61.0	0.78	2.98	70.0
Average	0.67	2.80	119.6	0.59	3.87	59.4	0.73	2.88	64.10
CoV (%)	12.2	20.3	23.4	26.6	34.2	15.2	11.3	10.7	15.6
FEM	0.61	2.86	108	0.55	4.2	73.1	0.79	2.32	65.5

**Table 8:** Test results: 30 min of creep investigation with a constant load of 18 kN.

Specimen	Type A			Type B			Type C		
	$\delta_{t=0}$ (mm)	$\delta_{t=30}$ (mm)	∆δ (%)	$\delta_{t=0}$ (mm)	$\delta_{t=30}$ (mm)	∆δ (%)	$\delta_{t=0}$ (mm)	$\delta_{t=30}$ (mm)	∆δ (%)
1	0.125	0.154	23.2	0.123	0.165	34.1	0.212	0.263	24.1
2	0.090	0.102	14.0	0.159	0.204	28.3	0.186	0.237	27.4
3	0.110	0.142	29.1	0.183	0.247	35.0	0.252	0.333	32.1
4	0.098	0.117	19.4	0.244	0.298	22.1	0.172	0.214	24.4
5	0.105	0.123	17.1	0.150	0.188	25.3	0.211	0.261	23.7
Average	0.106	0.128	20.4	0.172	0.220	29.0	0.207	0.262	26.3
CoV (%)	11.2	14.4	26.3	23.8	21.4	17.1	13.2	15.3	12.1

**Table 9:** Cohesive behavior parameters in the different models.

Cohesive Behavior Parameters [N/mm^3^]	Type A	Type B	Type C
K_nn_	1000	1000	1000
K_ss_	38	24	18
K_tt_	38	24	18

**Table 10:** Damage criterion parameters for all the models.

Damage Criterion Parameters [MPa]	
σ_nn_	5000
τ_ss_	1.5
τ_tt_	1.5

## Data Availability

Restrictions apply to the availability of these data. They may be individually shared at reasonable request due to [privacy reason].
